# School nursing in Germany - a developing field: results of a mixed methods study

**DOI:** 10.1186/s12889-025-25493-z

**Published:** 2025-12-11

**Authors:** Jana Kaden, Birte Berger-Höger

**Affiliations:** Department Evaluation and Implementation Research in Nursing Science, Institute for Public Health und Nursing Research, University of Bremen, Universitätsallee 1B, D- 28359 Bremen, Germany

**Keywords:** School health services, School nursing, Comprehensive nursing, Public health, Network, Nurse's role

## Abstract

**Background:**

School nursing is a well-established part of school health services globally. In Germany, school nursing is limited to a few model projects. Little is known about the school nurses’ role, its related tasks and goals, and the collaboration with interest-holders. The aim of the study is to explore to what extent and how school nursing is currently implemented in Germany, to provide a basis for further development.

**Methods:**

We conducted a context analysis, guided by the UK Medical Research Council’s framework from December 2022 to June 2023. A convergent mixed-methods design was used to gain a comprehensive understanding on the role, needs, working environment, and goals of school nurses. Quantitative data were collected via an online-survey and analysed descriptively. Semi-structured, guided focus group interviews, non-participatory observation of school nurses and expert interviews with interest-holders were conducted and analysed via content analysis for qualitative data, and typology formation was carried out. The findings were subsequently triangulated.

**Results:**

We analysed 65 survey datasets, qualitative data from five participatory observations, two focus group interviews, and ten expert interviews. School nurses work across various school types, caring for students of all ages. Of the respondents, 97% are registered nurses, with 34% holding a Bachelor’s degree. Their tasks, employment relationship and funding vary across federal states. Health promotion, prevention, and counselling are performed by 92%, addressing diverse topics depending on needs, 85% aim to strengthen health literacy among students, parents, and teaching staff. School nurses collaborate widely with internal and external interest-holders, are recognised as health experts and their work is valued supportive and accepted. Three different approaches for school nursing in Germany were identified. Using models and frameworks to inform school nursing was rarely reported.

**Conclusion:**

School nurses in Germany aim to contribute to students and their communities by health promotion, prevention and inclusion. Their role is multifaceted, combining nursing, education, and counselling with different emphases across schools and supporting broader public health efforts. When implemented, they reach students, school staff, parents and partly their community. Given its development stage, established international concepts could serve as a foundation and orientation for further implementation.

**Supplementary Information:**

The online version contains supplementary material available at 10.1186/s12889-025-25493-z.

## Background

School nursing is an integral part of school health or comprehensive school health services worldwide, for example the United Kingdom (UK), United States of America (USA), Canada or Scandinavian countries [1, 2]. School nurses serve as a bridge between health and education, aiming to enhance students’ educational and health outcomes by keeping the individual student healthy, safe, in school and ready to learn [2–5]. Integrated within the school setting, school nurses provide health expertise, help creating a healthy learning environment, and positively influence students’ health, wellbeing and academic outcomes [2, 4, 6]. Moreover, their expertise supports the promotion of students’ health literacy and the development of schools as health-promoting environments [7–9].

School nursing is a broad field and offered in different approaches, it includes public health offers and has the potential to reach students and their communities, both within and beyond the school setting [10–12]. In countries such as the UK or USA, school nurses play a central role in advancing the public health agenda by promoting health and addressing health inequalities within the school population [13–15]. Moreover, school nurses contribute to reducing healthcare disparities by addressing social and health determinants and facilitating access to healthcare services for (disadvantaged) students [14, 16]. They also provide health education, promote health literacy, and support school attendance to enable education [1]. Additionally, there is an increased need for nursing services at schools due to the rising number of students with complex needs and chronic conditions e.g. asthma, diabetes, life-threatening allergies, and medication requirements during the school day [17, 18]. Overall, school nursing is a complex clinical specialty practice [12], and includes public health nursing knowledge and skills [19] and partly primary health care, nursing, and social sciences [2]. Depending on the school nurse’s role, tasks, and resources, their presence at schools varies between full-time or part-time [2, 15, 20, 21]. School nurses’ qualifications vary internationally, ranging from a general Bachelor’s degree with additional training to Master’s degree in school, community or public health nursing, often complemented by prior experience in these fields [1].

The World Health Organization (WHO) strongly recommends the implementation of comprehensive school health services provided by qualified health professionals in primary or secondary education, addressing at least four of the following health areas relevant to the students: ‘positive health and development; unintentional injury; violence; sexual and reproductive health, including HIV; communicable disease; noncommunicable disease, sensory functions, physical disability, oral health, nutrition and physical activity; and mental health, substance use and self-harm’ [14]. There is moderate evidence from USA and other high income countries that (comprehensive) school health services are both cost-effective and cost-saving compared to schools with fewer or no such services [15, 22]. For successful implementation of comprehensive school health service, collaboration between the health and education sectors is crucial [14].

In Germany, the responsibility for schools and related topics lies with the education ministries of the federal states, while public health is organised on state, federal state and municipal level. The municipal public health authorities are entrusted with school health services as screening, school entry examinations or infection protection, and vaccination is predominantly provided by paediatricians or physicians. In recent years, pilot projects to implement school nurses have been launched in several federal states, some of which have been institutionalised and expanded [23, 24]. Early findings from these pilot projects highlight the positive benefits of school nursing on student health, cost effectiveness, and potential benefits for health literacy among students, parents, and teachers [8, 25]. Despite these advances, comprehensive data on school nursing in Germany is lacking, as there is no mandatory registration for nurses. Reports indicate a rising trend, with 64 school nurses reported in 2021 [23], and approximately 80 in 2022 according to the school health care network of the German Nurses Association (DBfK). Nursing qualifications in Germany are typically based on vocational training, and there is only one curriculum for a specialised training as “school health professional” [26]. Consequently, school nursing qualifications, organisational roles, and responsibilities vary significantly within Germany. Both community/public health nursing and school nursing are still in the development phases and have not yet been implemented nationwide. As such, little is known about the role of school nurses in Germany, their specific tasks, goals, and internal and external collaborations.

## Methods

The aim of the study is to explore to what extent and how school nursing is currently implemented in Germany, to provide a basis for further development of the field and subsequently to support the health of students inside and outside the school environment. Therefore, questions about the school nurses’ role and their practice, including barriers and facilitators should be answered, focussing on tasks, qualification, interprofessional cooperation, theoretical basis and goal setting for their work, target groups and work environment (funding, employment).

### Study design

This mixed methods study contributes to a context analysis of a larger project aiming to develop a complex intervention to promote the critical health literacy of school nurses in Germany. The related study protocol has been previously published [27]. Our whole project is oriented on the UK Medical Research Council’s (MRC) framework for the development and evaluation of complex interventions [28]. This context analysis on school nursing in Germany comprises an exploratory assessment to gain a comprehensive understanding on the role of school nurses, their needs and working environment and the aim of their work. We used a convergent mixed methods study design [29].

**Fig. 1:**
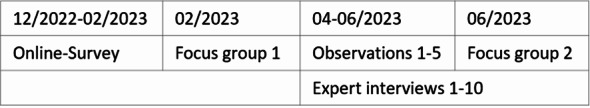
Timeline of quantitative and qualitative data collection

Quantitative data collection was carried out to provide a comprehensive overview. Based on these findings, qualitative methods were used to clarify, explain, complement and verify the quantitative data [30]. First an anonymous, open online-survey (S) of school nurses in Germany was conducted. Afterwards, we conducted qualitative semi-structured, guided focus group interviews (FG, focus groups) and open, non-participatory observations (O, observations) with school nurses. In addition, we interviewed relevant interest-holders as experts (E) on framework conditions, deployment and cooperation. Figure 1 describes the data collection of the study, rolled out from December 2022 to June 2023.

The study is reported following the Checklist for Reporting Results of Internet E-Surveys (CHERRIES) [31, 32] for the quantitative data and the Consolidated Criteria for Reporting Qualitative Research (COREQ) [33] for the qualitative data (Supplement 1 & 2). Additionally, it is guided by the criteria for Good Reporting of A Mixed Methods Study (GRAMMS) [34].

### Setting and sample

Due to historical developments and regional differences across federal states in Germany, various professional titles are used, such as “school nurse”, “school health professional” or “health professional at schools“(translated). In this study we use “school nurse” as a general term to encompass all related professional titles. The study targeted all school nurses in Germany, with inclusion criteria requiring current employment or employment within the last five years. We estimated approximately 80 school nurses exist nationwide. However, an exact count is unavailable. We tried to achieve the greatest possible participation in the online-survey. To capture the diversity of school nursing in Germany, we aimed to conduct at least one focus group and observe five school nurses with varying qualifications (e.g. vocational or academic training), working in different school types, settings, and federal states, and employing different approaches of school nursing. The focus groups were formed consecutively and homogeneously, based on the federal state in which participants work. For the expert interviews, we aimed to include at least five interest-holders related to school nursing, such as teachers, school directors, school social workers, employers or (former) project-partners, and international school nurses outside of Germany. The sampling was consecutive, guided by the results from the previously collected quantitative and qualitative data.

### Recruitment

The invitation with the link to the online-survey was sent via e-mail to employers, school nurses and the school health care network of the German Nurses Association (DBfK), who agreed to distribution, using snowball sampling method. The same recruitment strategy was applied for inviting participants to the focus groups and observations. Additionally, survey respondents could voluntarily provide their contact details if interested in participating in the qualitative phase of the study. Experts for the interviews were recruited through various channels, including contacts, networks, events, and the observations, using snowball sampling method. Participation in the study was voluntary, with no incentives or expense allowances offered. Ethical approval for the study was provided by the ethics commission of the University of Bremen (file number: 2022-17). All participants provided written informed consent prior to data collection. For the non-participatory observations, additional agreement of the employer and/or school director was required.

### Data collection methods

No validated questionnaires for the individual assessments were identified, therefore, all questionnaires and interview/observation guides were developed using the four step method [35], grounded in a literature review, and refined based on feedback from experts both within and outside the university setting.

#### Online-survey with school nurses

The anonymous open online survey consisted of 18 items covering school nurses’ tasks, qualifications, job duration, age-group, gender; setting (federal state and city size), school type, target groups, grades, number of students, applied school nursing model or approach (six open and 12 closed single or and multiple answers, partly with supplement option) (Appendix 1). The question on school nurses’ employment status was mandatory and served as a filter for the inclusion criteria. Additionally, questions regarding federal state, school type, grades, number of students and professional qualification were mandatory. Participants could review and modify their previous answers. Information about the study and data protection was provided before obtaining informed consent and starting data collection via LimeSurvey. Completing the two pages online questionnaire took approximately 30 min. Participants could pause and resume the survey using the same device, cookies were used to prevent multiple submissions. Two persons with public health/nursing degree pre-tested the survey regarding feasibility and comprehensibility, one of whom had prior experience as a school nurse. The survey was available for responses from December 2022 to February 2023, with a reminder sent via recruitment channels in January 2023.

#### Focus group interviews with school nurses

Semi-structured, guided focus group interviews (FG) were conducted to gain a deeper understanding, of what and how school nurses work. The interview-guide included questions on their tasks, teaching, evidence-based practice, educational needs, cooperation and acceptance, target groups, setting, school nursing models, funding, and barriers and facilitators in their work. Sociodemographic data collected included school nurses’ job duration, qualifications, age-group, gender, federal state, number of schools, school type, grades, number of students, and city size (Appendix 2). Sociodemographic data were collected anonymous, paper-based or via LimeSurvey. The focus group interviews with additional field notes were conducted face-to-face, in person or digital, by the author (JK) and a second researcher (LS).

#### Non-participatory observations

Open, non-participatory observations (O) of school nurses were conducted throughout an entire workday to gather insights into their work environment, tasks, interactions with students and school staff, and their overall practice. The observations, carried out by the author (JK), were guided by a structured observation guide covering school nurses’ tasks, teaching, evidence-based practices, educational needs, cooperation and acceptance, target groups, setting, school nursing models, funding, and the barriers and facilitators they face. Field notes were taken during the observations, and semi-structured follow-up dialogues were conducted to validate the observations (Appendix 3). Additionally, sociodemographic data similar to those in the focus groups, were collected by paper-based questionnaires.

#### Expert interviews with interest-holders

Semi-structured expert interviews with interest-holders were conducted (JK) digitally, via telephone or Zoom, depending on the expert’s preference. The open questions in the interview guide (Appendix 4) focused on barriers and facilitators of school nursing, the implementation of school nursing in Germany, future interventions, school nurses’ qualification, needs, and collaboration, tailored to each interest-holder’s role. Sociodemographic data, including the experts’ qualifications, job roles and their relation to school nursing, were also collected.

Qualitative data collection took place from February to June 2023 continuing until data saturation was reached. This was the case when sufficient information was gathered to answer the research questions and meet the research objectives. It was discussed collaboratively throughout the research process by the team. Interviews and observations were conducted flexibly, allowing for emergence of new themes and topics. Questions or information already covered were not revisited, instead, new questions were introduced as needed. All focus groups and expert interviews were audio recorded using an external device.

### Data analysis

Data analysis was conducted separately for quantitative and qualitative data (JK). Descriptive statistics were used for the quantitative - and sociodemographic data analysis in SPSS, Version 28.0.1. Audio recordings of the interviews were transcribed verbatim and anonymised, and paper-based notes were digitalised. All qualitative data, including the interview-transcripts, field notes, and open-ended responses from the online survey, were analysed using qualitative content analysis oriented on Mayring using a combination of deductive and inductive category development [36], with the qualitative software programme MAXQDA 2022 [37]. Two coding trees were developed, one for the expert interviews and another for all remaining qualitative data (Appendix 5). Initially, the coding trees were built deductively based on the topics from the interview/observational guides and online survey questions, with additional categories added inductively. The category systems were reviewed and refined within the research community before being finalised. The final category sets were applied to the entire data set in a subsequent analysis loop. All results were discussed within the research group. Additionally, the anonymised results of one focus group were validated through communicative validation [38–40] with one participant and a second school nurse team member. They confirmed the results, clarified specific aspects (e.g. complexity of network partners). Additional typology formation [41] was carried out to cluster different approaches of school nursing in Germany. These identified approaches were discussed and validated with national and international experts from the school nursing field.

### Data integration and triangulation

The quantitative and qualitative data were integrated at several stages. First, survey data were used to refine the interview and observation guides and to identify participants for the qualitative investigations. Second, the analysed quantitative and qualitative data were triangulated to combine and compare the results from the different investigations.

## Results

### Characteristics of the participants

#### Survey participants

A total of 71 school nurses participated in the survey, of whom six were excluded for not answering subsequent questions, leaving 65 valid data sets for final analysis. As incomplete questionnaires were included, percentage are based on the applicable sample size (*n*-value). Calculating a response rate is not possible, as the exact number of potential respondents is unknown.

School nurses from 12 of the 16 federal states of Germany participated in the survey (*n* = 64), all results are presented in Table 1. Two thirds of the participants (67%, *n* = 42) are employed in cities with 100.000 or more inhabitants. The majority of participants (98%, *n* = 62) identified as female. School nurses from all age groups participated, with 37% (*n* = 23) most aged between 25 and 35 years, followed by 29% in the 46 to 55 years age range (*n* = 18). Of 63 respondents, 97% (*n* = 61) held a professional nursing qualification. Additionally, 34% (*n* = 22) held a bachelor’s degree in nursing science, public health or a related field, and 16% (*n* = 10) specialised training as a „school health professional^1^“. 49% (*n* = 31) had worked as school nurses for one to five years, 28% (*n* = 18) for less than a year and 14% (*n* = 9) for over ten years. School nurses were employed across different school types. The majority (69%, *n* = 44) worked in primary schools (grade 1–4 or 1–6; depending on the federal state), followed by 16% (*n* = 10) at international schools. Depending on the school type and school nursing approach, some address students across the whole school period. 73% (*n* = 45) work at one school, 24% (*n* = 15) at two schools, and two on more than two schools (4 resp. 12). 36% (*n* = 21) are responsible for 500–999 students, 31% (*n* = 18) for 300–499, four for 1.500 or more students and three for less than fifty students.

**Table 1:** Participants’ socio-demographic data (online-survey, focus groups, observations)

	Online-Survey		Focus Groups 1&2 ^a)^		Observations ^a)^	
	*n*	%*	*n*	%*	*n*	%*
Federal states	64	*100 *	16	*100 *	5	*100 *
Number of different federal states	12		4		4	
Baden-Wurttemberg	3	*5*				
Bavaria	3	*5*				
Berlin	1	*2*				
Brandenburg	12	*19*				
Bremen	16	*25*				
Hamburg	4	*6*				
Hesse	9	*14*				
Mecklenburg Western-Pomerania	-					
Lower Saxony	1	*2*				
Northrhine-Westphalia	4	*6*				
Rhineland-Palatinate	9	*14*				
Saarland	-	*-*				
Saxony	1	*2*				
Saxony-Anhalt	-	*-*				
Schleswig-Holstein	1	*2*				
Thuringia	-	*-*				
City size - inhabitants	61	*100 *	16	*100*	5	*100*
>500.000	25	*41*	11	*69*	4	*80*
100.000 - 500.000	16	*26*	2	*13*	-	
50.000 - <100.000	5	*8*	-		1	*20*
20.000 - <50.000	6	*10*	3	*19*	-	
10.000 - <20.000	6	*10*	-		-	
5.000 - <10.000	3	*5*	-		-	
Age	63	*100 *	16	*100*	5	*100*
<25 years	1	*2*	-		-	
25-35 years	23	*37*	8	*50*	1	*20*
36-45 years	12	*19*	2	*13*	1	*20*
46-55 years	18	*29*	3	*19*	1	*20*
>55 years	9	*14*	3	*19*	2	*40*
Gender	63	*100 *	16	*100 *	5	*100*
female	62	*98*	15	*94*	5	*100*
male	-		1	*6*	-	
I do not want to categorise myself by gender	1	*2*	-		-	
Professional background *(multiple answers)*	63		16		5	
Geriatric nurse	1	*2*	-		-	
Paediatric nurse	26	*41*	4	*25*	3	*60*
Nurse	33	*52*	12	*75*	2	*40*
Bachelors' degree ^b)^	22	*34*	12	*75*	2	*40*
Specialist training ”school health professional“	10	*16*	3	*19*	1	*20*
Other ^c)^	22	*35*	6	*38*	2	*40*
Professional experience as school nurse	64	*100 *	16	*100*	5	*100*
<1 year	18	*28*	6	*38*	-	
1 - 5 years	31	*49*	6	*38*	2	*40*
6 - 10 years	6	*9*	3	*19*	2	*40*
>10 years	9	*14*	1	*6*	1	*20*
School type *(multiple answers)*	64	*100*	16	*100*	5	*100*
Primary school	44	*69*	15	*94*	4	*80*
International school	10	*16*	-		-	
School kindergarten/pre-school	8	*13*	1	*6*	-	
Gymnasium/grammar school	8	*13*	1	*6*	-	
Integrated comprehensive school	5	*8*	2	*13*	1	*13*
School with several educational programmes	4	*6*	1	*6*	1	*13*
Secondary school	4	*6*	-		-	
Special school	3	*5*	1	*6*	-	
Other types of school ^d)^	2	*3*	-		-	
Number of assigned schools	62	*100*	16	*100*	5	*100*
1	45	*73*	8	*50*	4	*80*
2	15	*24*	7	*44*	1	*20*
more than 2 ^e)^	2	*3*	1	*6*	-	
Number of students	59	* 100*	16	*100*	5	*100*
<50	3	*5*			-	
50 - 299	6	*10*	3	*19*	2	*40*
300 - 499	18	*31*	5	*31*	2	*40*
500 - 999	21	*36*	6	*38*	1	*20*
1.000 - 1.499	7	*12*	2	*13*	-	
≥1.500	4	*7*			-	
Grade level *(multiple answers)*	64	*100*	16	*100*	5	*100*
1. Grade	52	*81*	15	*94*	4	*80*
2. Grade	50	*78*	15	*94*	4	*80*
3. Grade	50	*78*	15	*94*	4	*80*
4. Grade	51	*80*	15	*94*	4	*80*
5. Grade	31	*48*	4	*25*	3	*60*
6. Grade	30	*47*	4	*25*	3	*60*
7. Grade	26	*41*	2	*13*	2	*40*
8. Grade	23	*36*	2	*13*	2	*40*
9. Grade	23	*36*	2	*13*	2	*40*
10. Grade	25	*39*	2	*13*	2	*40*
11. Grade	18	*28*	1	*6*	1	*20*
12. Grade	16	*25*	1	*6*	1	*20*
13. Grade	10	*16*	1	*6*	1	*20*
Other grade ^f)^	1	*2*	1	*6*	3	*60*

a) Due to anonymization requirements, participants sociodemographic data are reported in aggregated form

b) Bachelor's degree in nursing science, public health or a related field

c) Master in nursing science/public health, Anaesthesia and intensive care specialist, practice instructor

d) e.g. European school, “Oberschule” (secondary school), primary school grades 1-6; grades 1-13

e) 12; 4; 8

f) Vocational school students; Pre-class; Vocational orientation course

*percentage relates to the applicable *n* value and were italicized

#### Focus group participants

Two focus groups comprising a total of 16 participants (54/74 minutes) were conducted. The first focus group (FG 1) met face-to-face in the employer’s premises, while the second focus group (FG 2) participated online, via Zoom.

School nurses from four different federal states participated, with 50% (*n* = 8) aged between 25 and 35 years, and the remaining participants in older age groups. All participants were aged 25 years or older. 94% (*n* = 15) of participants identified as female, 69% (*n* = 11) work in cities with > 500.000 inhabitants. All participants hold a nursing degree, 75% (*n* = 12) hold a Bachelors’ degree and three completed a training as „school health professional“. 38%, each (*n* = 6) have professional experience of < 1 year or 1–5 years, *n* = 3 of 6–10 years and one > 10 years. Of the 16 participants, 94% (*n* = 15) work in primary schools. 50% each (*n* = 8 each) work at one school or at least two schools (Table 1).

#### Observation participants

Six school nurses from five different federal states were interested in participating in the open, non-participatory observations. However, one employer rejected participation, resulting in five observations conducted across four different federal states. The observations were conducted during the school nurses’ core working hours and lasted on average 7.2 h (range 5.5 to 8 h).

All participants were female, nurses, aged between 25 and 35 years or older. Two participants held a Bachelor’s degree or completed the training as “school health professional”. Two participants had 1–5 years of experience as school nurses, two had 6–10 years, and one had over 10 years of experience (Table 1). Three of the five participants worked primarily in primary schools. Four participants worked at one school, one at two schools.

#### Comparability of the participants “school nurse”

The qualitative investigation participants are mainly comparable to the survey participants, exception with 75% (*n* = 12) of the focus groups participants, academic qualified school nurses are over-represented, compared to 34% in the survey. None of the observation participants has professional experience less than one year. As in the survey also in the qualitative investigations most participants work at primary schools. No school nurse from international schools participated in the qualitative investigations. An overview of the whole demographics collected is presented in Table 1.

#### Expert interview participants

Ten experts were interviewed, with nine interviews conducted in German (five via telephone, one in person at the university, three via Zoom) and one in English (via Zoom). The interviews took in average 42 min (range 18–71).

The experts held various roles related to school nursing, including: teacher (*n* = 3), school director (*n* = 1), pedagogical assistant (*n* = 1), school social-worker (*n* = 1), project partner (*n* = 2), school nurse from another country (*n* = 1), and researcher (*n* = 1).

### Thematic results

Six main categories with their respective subcategories were built for the focus groups, observations, and open-ended survey responses: “School nursing model”, “Taks”, “Topics”, “School nurses‘ role and autonomy”, “Cooperation with other professions”, “General conditions school nursing”. In addition, six categories emerged for the expert interviews (Appendix 5). Data saturation was achieved for the research question.

### School nurses’ tasks

**Fig. 2:**
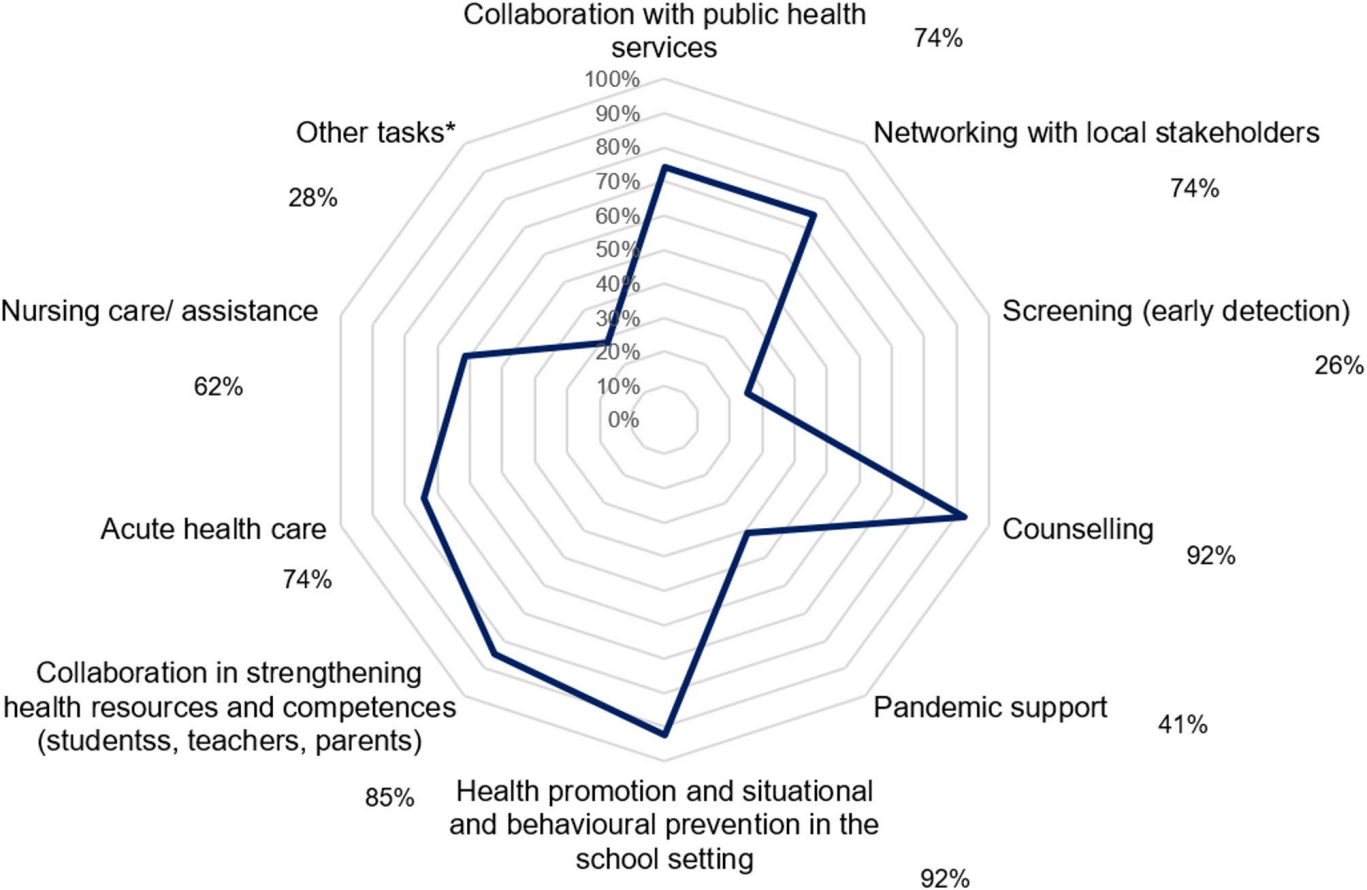
School nurses’ tasks, results of the online survey, multiple answers (*n* = 39)

A total of 39 survey participants reported their tasks (multiple answers, Fig. 2). Health promotion and prevention, as well as counselling, were offered by 92% (*n* = 36) of respondents. 85% (*n* = 33) collaborated to strengthen health resources and health literacy among students, parents and teaching staff. 62% (*n* = 24) rendered nursing care or assistance for students with chronic diseases. 26% (*n* = 10) conducted screening or early detection activities. Acute health care, including first aid, collaboration with local public health authorities, and networking with local interest-holders were all carried out by 74% (*n* = 29) of participants. Other tasks, selected by 28% (*n* = 11) included health education, parent involvement, administrative tasks, and health observation/support. 41% (*n* = 16) supported during the pandemic, this number is limited by a timing factor, as 29 survey-participants had been employed as school nurse for one year or less, likely starting in the final phase of the COVID-19 pandemic. Open answers and the qualitative investigations concretised school nurses’ tasks.

#### Health promotion and situational and behavioural prevention in the school setting

School nurses offer a range of health promotion interventions focused on healthy lifestyles, either during or after class (O 1–5, FG 1&2). The topics vary based on the needs and preferences of teachers and students, as well as the school nurses’ areas of expertise (O 1–5, FG 1&2). Common topics include nutrition, physical activity, the human body, first aid, addiction prevention, hygiene, relaxation and mindfulness, herbalism, skincare, mental health, bullying, grief coping; (digital) media use, road safety, and environmental issues. In their approach, school nurses integrate both theoretical and practical elements, with some incorporating physical activities during breaks (S, O 1–5, FG 1&2). School nurses work to create a healthy school environment by advocating for healthier food offers in the cafeteria, collaborating with parents, school-, and cafeteria staff and providing water and cups or bottles. They establish hygienic standards, such as for school kitchen, assess the school area for injury risks, and promote a health-conscious, physically active environment (O 1–5). No school nurse reported vaccinations as part of their tasks, (O 1–5, FG 1&2), though some checked students’ measles vaccination records on measles vaccination, as it is mandatory in Germany (O 4). School nurses primarily focus on primary prevention at the behavioural level, while secondary and tertiary prevention is provided to students with chronic conditions like diabetes or through screening exams (O 1–5, FG 1&2).

#### Collaboration in strengthening health resources and competences (students, teachers, parents)

School nurses play a pivotal role in health promotion within educational settings, working closely with students, teachers, and parents to enhance health resources and competencies (O 1–5, FG 1&2, E 4–7, 9, 10). Their responsibilities encompass providing health information, fostering a healthy lifestyle, and supporting the development of health literacy among all, students, school staff and parents (O 1–5, FG 1&2).

#### Counselling

Counselling is a central component of school nurses’ work, addressing the diverse needs of students, school staff and parents. These counselling activities are conducted individually or in group settings and cover a broad range of topics, including nutrition, healthy lifestyle, digital media use, and specific questions about disease management within and beyond the school environment (O 1–5, FG 1&2). For vaccination-related questions, school nurses primarily organise external resources or refer to healthcare professionals outside the school (FG 1, O 1–5). The scope of counselling is shaped by the needs of the school community. School nurses also serve as trusted points of contact, offering advice to teachers, teaching assistants, inclusion assistants, and parents.

#### Acute health care/First aid

The provision of acute health care varies across school nursing approaches in Germany, with all school nurses delivering first aid in emergencies (FG 1&2, O 1–5). Beyond basic first aid, some school nurses offer additional acute care for sick students. In these cases, students may seek assistance independently or be referred by the teachers. The school nurse assesses whether the student should remain at school or go home, informing both, parents and teachers as necessary. For chronically ill students, some school nurses maintain on-site supplies of glucose for diabetics, or personalised emergency medications, which they are authorised to dispense (O 3–5). These nurses also provide age-appropriate education for students on managing their symptoms and illnesses when consulted (O 3–5, FG 2). Additionally, school staff frequently seek advice from school nurses for health-related concerns (O 3–5). However, this extended scope of acute care is not universally implemented, depending on the school nurse’s designated responsibilities, and related to the task is necessary equipment. Some school nurses extend their role to first aid education, offering training for students across different grades (FG 1&2, O 1–5). Others have initiated specialised courses for school staff, addressing their individual needs within and beyond the school setting, or established a student’s school medical service (FG 2, O 4&5). These initiatives often involve collaborations with local aid organisations, such as the German Red Cross (DRK).

#### Nursing care/assistance

Supporting students with chronic conditions, special needs, or health challenges resulting from prolonged illnesses is a crucial aspect of school nurses’ responsibilities. To achieve this, they support case- and care management, provide education to the students, their families, the classmates and school staff about the disease, its management and how to deal with related emergencies during the school day (O 3–5, FG 2). This comprehensive support fosters a sense for security among all parties involved and promotes the inclusion of affected students, as emphasised by school nurses (O 3–5).

#### Pandemic support

School nurses provided essential support in organising and implementing testing procedures, enforcing hygienic measures, and advising on health protocols and concepts (O 3–5).

#### Screening/early detection measures

Some school nurses conduct vision and hearing tests of students as part of the public health–organised school examinations in grades 3 and 6. (O 3,5, FG 2). By contrast, at schools serving the Danish minority in Germany^2^, school nurses conduct health examinations autonomously at school entry and in grades 3, 6, and 8.

#### Collaboration with public health services

Next to the support of school screening examinations, school nurses are closely connected to the public health authority, or employed within it, receive information following school entry screenings to address or prepare for students’ future needs (O 1–5). Some collaborate directly with the health authority staff, including the child and youth health service (KJGD) or dental services (ZÄD). These collaborations may involve regular meetings (e.g. annually), professional guidance on medical or health-related questions, and joint efforts on health promotion and hygiene initiatives (O 3–5, FG 2).

#### Networking with local interest-holders

School nurses establish an individual support network to address students’ health needs, both within and outside the school community (e.g. parents, teachers, school-social-worker, cafeteria staff) and externally, with local partners, organisations and institutions (police, health insurance, physicians) who provide financial or direct support (O 1–5, FG 1&2). Some school nurses develop a support network for parents and students to assist them with health-related matters, such as psychologists, doctors, therapists, or aid organizations (O 1–5). Some try to build a network with other health actors for health promotion offers in the community (FG1, O1&2).

#### Health education

Health education and teaching support is an additional category of responsibilities for school nurses. All focus group and observation participants reported involvement in group-based health education, which is closely tied to health promotion and topics such as first aid (S, O, FG, E 4, 6–8). Therefore, school nurses support teachers and other school staff (school social worker) by co-teaching, offering professional advice or collaborating on project work. These activities are carried out either independently or with internal or external partners. In terms of sustainability, school nurses address health topics over several years, ensuring continuity, and are closely associated with these issues by the students. In practice, school nurses communicate their health education offerings to teachers, who then decide whether to incorporate them into the curriculum or not. In general ideas and topics to address originate from both sides, the school nurses and teaching staff. The offers are well used by a part of the teachers, but not by all (O 1–5, FG 1&2, E 4). Some school nurses face challenges in providing health education in practice due to a lack of pedagogical qualifications, especially when teachers are absent from the classroom. In general, teachers need to be in class to support during the lessons (O 1–2, 4, FG 1).

#### Other tasks

In addition to their primary responsibilities, school nurses also handle administrative tasks, including daily reporting on their activities, which is mandatory for some (FG 1, O 1&2). They organise projects, participate in school working groups, and promote parent engagement.

Overall, many of school nurses tasks are interconnected and cannot be considered separately. No systematic needs assessment by the school nurses was reported. In addition to meeting the specific requirements set by their employers, and addressing identified needs by school staff and students, the tasks undertaken by school nurses are influenced by their individual qualifications. Nurses typically focus on topics with which they are most familiar, as there is flexibility in how tasks and activities are designed and implemented (FG 1&2, O 1–5).

Table 2 presents example quotes and qualitative observations from the school nurses (O, FG) to the presented tasks.

**Table 2:** School nurses’ tasks - example quotes from the qualitative investigations

**Health promotion and situational and behavioural prevention in the school setting**
- *Examples for the topic nutrition: the school nurses explain the food pyramid and provide healthy food activities*,* purchase the food together and/or prepare meals together with students. Therefor they cooperate with school internal and external partners in kind of funding and/or personnel support (O 1&2).*
- *At the children’s request*,* a punching wall was installed in the hallway for them to release tension (O 2)*
- *„Even if it’s about the topic*,* for example*,* that the school staff somehow need something for their health*,* yes*,* and maybe they want a space to rest and relax*,* a place to retreat*,* then that’s not feasible because there’s simply no room for it*,* so things like that…that’s just a pity somehow” (FG1: 108)*
**Collaboration in strengthening health resources and competences (students, teachers, parents)**
- *“Health literacy*,* in this context*,* cannot simply be imposed; it is a process of development*,* including self-awareness. This means that one must continually provide impulses and consistently communicate it to people*,* whether they are children or adults*,* parents or teachers.” (FG1: 52)*
- *“Well*,* for us*,* regarding health literacy*,* it’s also my task to look at what is possible for the students. Just like [S1] also mentioned*,* when they come with a stomach ache or a headache*,* the pain is there*,* but what could be the reason? […] Because they know what is actually wrong with them. Or they also have an idea of what it might be related to. And then I don’t have to dictate everything*,* but instead guide them a bit through questions*, etc.,* to get them on the right track or to help them think for themselves. So*,* I think that is really my main task.” (FG2: 76–80)*
**Counselling**
- *„Well*,* for us*,* for example*,* the focus has been very much on nutrition and physical activity—like children who might be overweight or*,* in some cases*,* very underweight*,* which I’ve also encountered. Additionally*,* there are children who eat unconsciously*,* often in large portions*,* without really reflecting on what they’re consuming. Media consumption is another major issue; parents often report that it’s challenging to get their children away from screens. In many cases*,* media is used almost as a way to keep them quiet. These kinds of problems are frequently brought to my attention by parents.” (FG1: 46–47)*
- *“Trusted contact person*,* meaning anything brought to me by teachers*,* teaching assistants*,* or inclusion assistants that involves a need for advice. Or from parents—it really comes from all directions.” (FG2: 29)*
**Acute health care/First aid**
- *“The children are not sent to us if they have simply cut themselves or need an ice pack*,* because we are not on-site at the school every day. In these cases*,* the teachers take care of it.” (FG1: 12)*
- *“Acute care*,* where I assess what the students need in the current situation—whether it’s stomach pain […]*,* a fall*,* wound care*,* and so on—these tasks occupy a considerable amount of the day.” (FG2: 29)*
**Nursing care/assistance**
- *“This care for students with chronic conditions falls more within our scope of activity in terms of providing advice*,* if needed*,* but we are not responsible for the direct care of these students.” (FG1: 12)*
- *The school nurse reported*,* to advise teachers and the class*,* after prior consultation with the parents*,* on what to do in case of an emergency (e.g.*,* epileptic seizure). Emergency medication is either with the school nurse or the student. In many cases*,* the class is able to handle such situations very well*,* and the children can participate in the class community as usual*,* outside of these emergency situations. This provides reassurance to parents*,* children*,* and teachers. The children perceive this as positive*,* as they do not feel defined by their illness*,* and the condition becomes irrelevant in everyday life. (O 5)*
**Pandemic support**
- *An example reported was the mask mandate*,* as a few wanted to disregard it*,* and those few kept the school nurses very busy: „A few can occupy you“ (O 5)*
**Screening/early detection**
- *“[We] also examine children regularly. We examine them when they start school*,* often when they enter kindergarten*,* then when they start school. Then we see them in third*,* sixth and eighth grade. And then the sports students who take sport as a profile*,* we also examine them. […] school doctor tasks plus the Danish tradition […]. And during the tests*,* we do hearing and eye tests. We measure and weigh them“ (FG2: 10)*
- *Screening/school examination in year 3 by school nurse alone*,* in preparation information is given to parents for consent by the public health authority; eye test*,* hearing test*,* height*,* weight by school nurse*,* recommendation and information to parents in case of findings (O 3)*
**Collaboration with public health services**
- *After school entry examination*,* the school nurse receives information if children with special needs and information to parents through KJGD*,* if agreed*,* then KJGD establishes contact between school nurse and parents*,* e.g. obesity*,* ADHD*,* metabolic disorder (O 3)*
- *Hygiene (e.g. lice) cooperation with public health department/health inspectors (O 3*,*4)*
**Networking with local interest-holders**
- *For project funding e.g. school and community together with adult education center*,* as they can apply for funding*,* school nurse is not able to do this (O 2)*
- *Cooperation with the rural women’s association that supports the nutrition certificate*,* this is also supported by the health insurance company (O 1)*
**Health education**
- *“Since I’m now in classes quite a lot*,* I usually take over the lesson for a full double period*,* with the teachers mostly just being present and rarely getting involved. There’s also a lot of organization involved*,* like bringing external projects into the school.” (FG1: 9)*
- *“I teach a 10th-grade on health*,* which I’ve been doing for a year now. It’s also something that should be further developed for 9th-grades.” (FG2: 29)*

### Cooperation and acceptance of school nurses

Both, school nurses and school staff reported broad acceptance and positive recognition of the school nurse’s role and tasks within the school community, including by students and parents. Teachers view school nurses as valuable health experts and support resources, though their offerings are utilised differently depending on the individual teacher’s needs. From the school nurses’ perspective, offers tailored to the needs of students’ and the school community ensure successful implementation and acceptance. The cooperation between school nurses and school staff perceived as positive and supporting from both perspectives (FG 1&2, O 1–5, E 4,6,7,9). One perception was:

*„So*,* what I gathered was that during the COVID period*,* she [the school nurse] actually went into the classrooms and explained to the students again about social distancing and so on. And*,* of course*,* in that context*,* she had a completely different standing*,* because she’s the health expert. Teachers*,* of course*,* are always associated with rules and enforcing those rules from the students’ perspective. I think this made it much easier for her message to be received in a different and much more open way by the students. She’s the expert in that field*,* and in her role*,* she’s not involved in things like grading or assessing academic performance*,* so she’s not in that typical teacher role.“ (Exp9: 25)*.

Clearly defined roles and tasks between teachers, school social workers and school nurses enhance collaboration (O 1–5, E 1–7, 9). Teachers and school staff feel relieved by the support of school nurses and expressed a desire to expand these serviced to more schools, particularly for (acute) health care for sick children. They appreciate the nurses’ contribution to students, including their participation in working groups, which are seen as enriching and beneficial for students’ development. (E 2,4,6,7,9). A teacher described:

*“Well*,* I really think it’s very*,* very important that every school has a school nurse. One who*,* like ours*,* plays such an important role […] I’m truly very*,* very thankful for that […] I would never want to miss our school nurse […] I actually wish this for every school*,* because I can really see in my interactions with the students*,* throughout their whole school time*,* even with the student first aid team […] it’s so important that we have this. It has such a positive impact on the students.” (Exp7: 87)*.

Expanding school nurses’ responsibilities to include acute health care, alongside their primary focus on health promotion, prevention, and group activities, would be welcomed by school staff. However, it is emphasized that existing tasks remain vital and should not be reduced or replaced for this purpose (E 4).

### Qualification

The qualification as a nurse is required in most federal states, in some additional a Bachelors’ degree in nursing or public health or a related field is mandatory. Others require training as a school health specialist, aligning with the specific tasks of the role (FG, O, E).

### School nursing approaches in Germany

Three school nursing approaches in Germany were identified based on the data collected, and verified with employers, school nurses and interest-holders (Table 3). These are called by the researcher (1) Holistic school nursing approach, (2) Health promotion approach, and (3) Danish school nursing approach. Common to all is health promotion and prevention, counselling and health education, performed with different focal points. Enabling school attendance as a goal or overarching task of school nurses is a central difference between the holistic school nursing and health promotion and prevention approach.

**Table 3:** Comparison of school nursing approaches in Germany based on results of the mixed-methods study*

Approach*	Holistic school nursing	Health promotion	Danish model
Aim/goal	ensuring stable school attendance and learning	strengthening students’ health resources and health literacy	health promotion and prevention with early detection
Student-focus	individual & groups	groups	individual and groups
School type	all school types	primary schools (grades 1–4)	primary and secondary schools
Presence at school	daily	not daily	not daily
Federal states	a. o. Baden-Wurttemberg, Brandenburg, Hesse, Rhineland-Palatinate;	Bremen, Hamburg	Part of Schleswig-Holstein
Tasks			
Acute health care/First aid	x	(x)^1^	(x)^1^
Collaboration with public health services	x	x	x
Counselling	x	x	x
Networking with local interest-holders	x	x	(x)
Health education	x	x	x
Health promotion and prevention	x	x	x
Nursing care/assistance	x	-	-
Pandemic support	x	x	x
Screening	(x)^2^	-	x
Strengthening health resources and competences/literacy	x	x	x

(x) partly

^1^ first aid in case of emergencies, if on-site

^2^ support of the screening examinations of the public health authority

*The three different approaches were labelled from the research group

1. Holistic school nursing approach focus on the individual student, providing (acute) care and counselling, and supporting case and disease management for the individual plays a major role. It also includes health education activities and counselling for groups. Overall, nearly all of the tasks reported above are included in this approach, except for some screening tasks. These professionals call themselves school nurse or school health professional. The approach is provided in different federal states and school types and the training qualification „school health professional” focus on this approach. They have the appropriate spatial and material resources available to provide acute and nursing care. The goal is ensuring stable school attendance and learning, and school nurses tasks focus a. o. on this: *„as our principle [goal and related tasks are] to enable a child*,* regardless of their condition*,* to have a positive school day or a successful school attendance.” (FG2: 84)*.
2. Health promotion approach which is primarily group oriented. It is more a public health approach focusing on health promotion, prevention, health education and health literacy; network tasks and counselling. *“Strengthening the health resources of the students*,* that’s kind of a main focus*,* I would say that this is also somewhat the goal of our work.”* (FG1: 5). The professional title is “health professional at schools” and it is provided in two federal states, Bremen and Hamburg. The goal/aim is to strengthen health resources, competencies and health literacy and to address health inequalities.
3. Danish school nursing approach, provided at Danish minority schools in the federal state Schleswig-Holstein with the aim of early detection, health promotion and prevention. The school nurses’ main focus is on monitoring the children’s and adolescents’ health regularly throughout the school years [42, 43]. As part of a school health team, school nurses conduct screening examinations, health promotion and prevention, counselling and health education.

### School nurses role development and practice implementation

The structure and tasks of school nursing vary across Germany, with state-specific frameworks established primarily in federal states with current or former model projects. Funding and employment models differ, such as the Ministry of Education in Hesse, public health department in Bremen and non-public funding at private schools (FG, E, O). In some states, implementation is guided by a needs assessment, prioritising schools in socioeconomically disadvantaged districts and/or high needs assessed in school entry health screenings (FG 1, O 1–4). School nurses report autonomy within the defined task frameworks (O 1–5), developing their roles based on “best knowledge” and work shadowing (O). While this flexibility is valued, it can be challenging to maintain boundaries and avoid task overload from school staff. Others benefit from structured task development during pilot projects or through school health training programs. Professional exchange opportunities vary widely, from weekly meetings to quarterly sessions, depending on the federal states (FG, O, E 3). However, school nurses in private schools or isolated roles often lack access to such networks, making it difficult to seek professional reassurance or advice. As described by an expert:

*“The school nurses are*,* so to speak*,* very much lone fighters at the school […] they don’t work as they are used to*,* perhaps from their previous work in a team*,* they work alone.” (E 3: 95–99).*

This lack of peer support was reported as a challenge by both school nurses and experts (O 3–5, E 1,3,5). Networking among school nurses and the expansion of state-level initiatives or the school health care network of the DBfK, are considered crucial for advancing the still-developing field (E 1,3,5, O 3–5).

School nurses covering multiple schools expressed a preference for focussing on a single school, which would allow for improved networking, better time allocation, and expanded offerings for students, staff, parents, and the broader community, often in collaboration with local partners and networks (O 1,2; FG 1&2). As reason given was a lack of financial resources for more staff.

The availability of dedicated office space and medical equipment varies by approach. While some nurses have their own office and proper equipment to provide medical care, others share space without access to specialised tools (FG, O, E):

*„Our offices are not equipped for that*,* […] we do not have a treatment couch or anything in our office where we could attend to injured children – this is outside the scope of our responsibilities.” (FG1: 15)*.

*„There are certain room requirements that a school needs in order to provide a suitable workplace for the school health professional.“ (Exp3: 23–27)*.

### Models and frameworks in practice

27 survey-respondents described the school nursing models they use in daily practice. Responses were grouped into three categories: “*International concepts/models*”, “*School nursing projects curriculum Germany*” (oriented on the training curriculum, developed to qualify school nurses in Germany) “*Activity/task description/objectives*”. One respondent referenced international models, three mentioned the German school nursing curriculum, and 23 focused on task descriptions or objectives. The reported objectives included promoting equal opportunities, strengthening health resources, improving health literacy, preventing illness, enabling participation and inclusion, reducing school absences, and minimising external medical appointments through onsite care:

*“The aim is to optimise equal opportunities in education/to promote educational participation.” (S 85: 5).*

Four observed school nurses reported not using a specific model, whereby two of them are oriented on international school nursing role models and one incorporates different models:

*„No direct model*,* but various models are incorporated into the work*,* such as salutogenesis*,* health promotion and prevention“ (O 2:37).*

Some school nurses reported strengthening of students’ health resources as goal of their work, others’ aim is facilitating school attendance for all students (FG 1&2). Others highlighted the role of task descriptions in framing their work, including providing legal protection for responsibilities like advanced medical or emergency care (FG, O 4,5).

## Discussion

The study reveals diverse school nursing approaches in Germany, supporting students, families, and school staff across all school types, but mainly on primary schools. When implemented, school nurses address students’ health needs and provide health promotion and prevention throughout their school years. Given the declining health trends among children, evident in rising rates of overweight, dental problems, and mental health disorders [44–46], school nurses are uniquely positioned to contribute to sustainable health promotion and the well-being of children. At the time of data collection approximately 80 school nurses were employed in Germany, compared to 32.758 general education schools [47]. Currently, health is not a teaching subject in Germany, compared to other countries e.g. Canada or Finland [48, 49]. As a result, school nurses rely on the teachers’ willingness to integrate their health education offers into different lessons. While practice examples indicate that this integration is feasible across different subjects, introducing “health” as a formal teaching subject could foster greater commitment. School nurses could be supported in delivering health education and promoting health literacy by centrally provided, evidence-based materials. Their collaboration with public health authorities and health provider supports closing existing gaps caused by segregation by bundling information or tasks. This approach enables early interventions and ensures stable health services for students.

There is no standardised approach, funding or employment framework for school nursing in Germany, due to the federal structures and historical development often as individual initiatives or as (model) projects. The data reflect both, the development stage of the professional field, and the long-standing implementation of offers, for example in international- or private schools. School nurses with highest nurse to student ratio are not necessarily employed at multiple schools, depending of the approach, working at more than one school in a full-time position is often a basic requirement. Nurse to student ratios differ internationally from around 1:125 to 1:750 − 1.200, depending on the students’ health care needs [21, 50, 51]. Lower school nurse-to-student ratios are associated with improvements in health and educational outcomes for students with chronic conditions [21]. Future implementation efforts should consider the complexity of workload including factors related to students, school nurses, schools and communities [51]. The National Association of School Nurses (NASN), recommends the daily presence of a registered school nurse to optimise students’ health, safety and learning [52]. This recommendation is based on the Whole School, Whole Community, Whole Child model [53] and the “Framework for 21 st Century School Nursing Practice”, updated 2024 “School Nursing Practice Framework™” [3, 54].

All school nursing approaches provide comprehensive school health service according the WHO definition [14]. Considering the top five school health interventions international [55], nutrition screening and vaccinations are not offered; whereby sexual and reproductive health education, nutrition health education and partly vision screening are offered by school nurses in Germany. Interventions with reported evidence of effectiveness for conditions such as autism, depression, anxiety, obesity, dental caries, visual acuity, asthma, and sleep [22] are partly provided. A shared goal across all approaches is to improve students’ health and health literacy while addressing health inequalities by offering health promotion, prevention, counselling and health education. School nursing is understood as a specialised nursing practice that promotes students’ well-being, school attendance, academic success, lifelong achievement and health [3, 5, 21]. It goes beyond public health approaches in health promotion and prevention and includes direct care for students [1]. This is established in the holistic school nursing approach, only.

The identification of the three different approaches is a first step, to explain school nursing in Germany. A wider range of school nursing models and frameworks is established international. Compared to them, the identified holistic school nursing approach includes elements of holistic school nursing [56] and the Framework for 21 st century school nursing practice [12]. Elements of health promotion approach are comparable to comprehensive school health in Canada [2]. The WHO provides no specific recommendations about the preferred scope of school health service due to a lack of evidence for the effectiveness of the different interventions [14]. As in other countries [14], Germany faces intersectoral challenges resulting from different responsibilities and different employer for school nurses by either education or health sector. The advantages and disadvantages of both are actually discussed in Germany. School nursing in Germany was positive evaluated during model projects [8, 25] and has the potential for nationwide implementation in all school types if appropriate resources are provided. Criteria for school nurse implementation need to include, that health care offers or access for disappeared students could only be provided by school nurses when implemented in appropriate areas [16].

### Theoretical framing

The overall picture shows a model or theory as basis for school nurses practice in Germany is rarely reported, school nurses mainly reported their tasks and the goal of their work, no standardised model is established in Germany yet. Reasons for this may lie in the different approaches and goals of the model projects, related job requirements were more task oriented, the partly non-academic qualification and the fact that the field is not yet well developed. The own role development partly based on an inductive approach without professional external support, role models or additional specific qualification is lacking, especially in single implementations of school nursing outside from model projects or international connections. Predominantly task orientated work entails the risk of being limited in scope [57] and a lack of international compatibility. School nurses mostly work alone in their school, there is a need for orientation and delimitation a framework, model/theory might support them, strengthen role clarity, evidence-based practice and communication with third parties [1, 4]. Further steps could include a common goal setting for school nursing research priorities [58].

No standards or legal regulation regarding school nursing in Germany exist, resulting in a lack of a minimum requirements or qualifications to hold the professional title school nurse and related tasks. This entails the risk of deprofessionalisation and quality loss, by employment of insufficiently qualified staff. However, the actual results show that nearly all participants are qualified as nurse. Even the qualification international varies, the minimum requirement typically includes a Bachelors’ degree [1, 2]. Related to their tasks in health education an equivalent qualification level to teachers is recommended [59]. During school nurses tasks are international differentiated to bachelor, master or doctoral level [60], no one reported operation on policy level, nor a differentiation of school nurses tasks between education level or working with e.g. nursing assistance. Developing of these structures might support further implementation of school nursing in Germany. School nurses are confronted with complex situations and needs in their daily practice. To address these challenges, academic qualifications and competencies regarding the different approaches should be pursued.

### Strengths and limitations

A strength of this study lies in the mixed methods approach, which allowed the combination of various data sources to examine the diverse group of school nurses and interest-holders in Germany, providing a comprehensive overview of the current state of development and implementation. The triangulation contributed to gaining a comprehensive understanding from multiple perspectives. However, the view of the students and/or parents on the school nurses’ work is missing in our exploration. For feasibility reasons we focused on the professional side. We consider these perspectives important and thus they should be included in further research in context of school nursing in Germany. Calculating of a response rate for the online-survey is not possible due to a lack of statistics on school nurses in Germany. However, we employed various recruitment methods to ensure a broad distribution of the survey. Based on previous estimates, we assume a relatively high response including participants from all school types. Cookies were used to minimise multiple participations and associated bias; further measures were not possible due to data protection constraints. School nurses across the different investigations are predominantly comparable. A limitation of the qualitative data collection is the potential for self-selection bias, as participants predominantly reported positive experiences, without mentioning challenges related to acceptance or roles/tasks. Participants in the observations noted that such issues, while rare, do occur. School nurses from international schools could only be recruited for quantitative data collection, which may result in a lack of information, particularly regarding the different school nursing approaches in Germany. All research was conducted by one researcher (JK) with a background in nursing science and public health. To minimise the limitation of data analysis conducted by one researcher, a continuous discussion of the single elements with other qualitative researchers of the research community of the university, and a qualitative validation of results with participants was carried out.

Further research is needed to identify barriers for success of a nationwide implementation of school nurses as well as a sustainable financing concept, for which international models can also provide orientation [1].

## Conclusions

Our study provides a data-based overview on school nursing in Germany, which could support further development and implementation efforts. The role of school nurses is highly diverse, they act as nurses, educators and counsellors with varying emphases depending on the school context, and they contribute to public health. When implemented, they reach students, school staff, parents and partly their community. Despite differences in their approaches and focus areas, they have the potential to play a key role in improving students’ health and supporting education. However, compared to international school nursing concepts, Germany lacks a robust theoretical foundation. Given its development stage, established international concepts could serve as a foundation and guide for orientation. Further research is needed to develop a shared understanding and clear objectives for school nursing in Germany, as well as to define the required qualifications and to foster practice development. Those involved and responsible for this field should continue to collaborate and advance these efforts.

## Supplementary Information

Supplementary Material 1. CHERRIES-checklist.

Supplementary Material 2. COREQ_Checklist-SchoolNursing-BMC.

Supplementary Material 3. Appendices 1–5.

## Data Availability

The datasets used and/or analysed during the current study are available from the corresponding author on reasonable request.

## Abbreviations

CHERRIES: Checklist for Reporting Results of Internet E-Surveys
COREQ: Consolidated Criteria for Reporting Qualitative Research
DBfK: German Nurses Association
DRK: German Red Cross
E: Expert interview(s)
FG: Focus group interviews
GRAMMS: Good Reporting of A Mixed Methods Study
KJGD: Child and youth health service
MRC: United Kingdom Medical Research Council
NASN: National Association of School Nurses
O: Open, non-participatory observations
S: Open online-survey
UK: United Kingdom
USA: United States of America
WHO: World Health Organization
ZÄD: Dental services

## Footnotes

1 The special 10-month-training “school health professional“ addresses school nurses needs in their daily practice on competence level 6 (DQR/EQF), but it is not a bachelor’s degree.

2 The Danish minority consist of persons living in the northernmost German and live their lives as Danish as possible, for example with Danish nurseries or schools.
