# Supplementary material for: School nursing in Germany - a developing field: results of a mixed methods study
Source: BMC Public Health. 2025 Dec 11;26:196. doi: 10.1186/s12889-025-25493-z (PMC12801880; doi:10.1186/s12889-025-25493-z)
Supplement: Supplementary file 3 — Supplementary Material 3. Appendices 1–5. [file 12889_2025_25493_MOESM3_ESM.pdf]

Appendices for
School Nursing in Germany - A developing Field: Results of a Mixed Methods Study

Jana Kaden, Birte Berger-Höger

Content

Appendix 1. Survey questionnaire* ... 1
Appendix 2. Interview guide focus groups* ... 3
Sociodemographic data focus group and observations* ... 4
Appendix 3. Observation guide* ... 6
Appendix 4. Interview guide expert interviews, example ... 7
Appendix 5. Final category sets ... 8

Supplement 1 CHERRIES checklist (separate PDF)

Supplement 2 COREQ checklist (separate PDF)

*Note: The questionnaires used in the study were in German language. For reasons of transparency, they are translated in English, these versions were not tested or validated.

School Nursing in Germany - A developing Field: Results of a Mixed Methods Study
Jana Kaden, Birte Berger-Höger

Appendix 1. Survey questionnaire*

Question	Answer category	Mandatory
Are you currently or have you been working as a school nurse (incl. school health nurse, school health professional or other title) in Germany in the last 5 years?	yes<br>no	yes
Note: Since there are different job titles in Germany, such as school nurse, school health specialist, school health nurse, health professional at schools, and others, the term "school nurse" is used in this survey for simplicity, and it also refers to all other titles. If you have worked as a school nurse in the past, the following questions refer to your latest position as a school nurse.		
Questions about your personal background and professional experience		
How long have you been working as a school nurse?	☐ less than 1 year<br>☐ 1 - 5 years<br>☐ 6 - 10 years<br>☐ more than 10 years	no
Which age group do you categorise yourself in?	☐ younger than 25 years<br>☐ 25-35 years<br>☐ 36-45 years<br>☐ 46-55 years<br>☐ older than 55 years	no
Which gender do you identify with?	☐ female<br>☐ male<br>☐ inter*<br>☐ trans*, transman, transwoman<br>☐ An identity not mentioned here.<br>☐ I do not want to categorise myself by gender.	no
In which federal state(s) do you carry out your professional activity as a school nurse? (multiple answers possible)	☐ Baden-Wurttemberg<br>☐ Bavaria<br>☐ Berlin<br>☐ Brandenburg<br>☐ Bremen<br>☐ Hamburg<br>☐ Hesse<br>☐ Mecklenburg Western-Pomerania<br>☐ Lower Saxony<br>☐ Northrhine-Westphalia<br>☐ Rhineland-Palatinate<br>☐ Saarland<br>☐ Saxony<br>☐ Saxony-Anhalt<br>☐ Schleswig-Holstein<br>☐ Thuringia<br>☐ other, namely:	yes
In how many schools do you work as a school nurse at the same time?	☐ 1<br>☐ 2<br>☐ more than 2, namely:<br>☐ no answer	no
The school(s) in which you work is/are: (multiple selection possible)	☐ School kindergarten/pre-school<br>☐ Primary school<br>☐ Gymnasium/grammar school<br>☐ Special school<br>☐ Integrated comprehensive school<br>☐ School with several educational programmes<br>☐ Secondary school<br>☐ International school<br>☐ other, namely:	yes

Appendix 1.Survey questionnaire* cont.

In which grades do you look after children? (multiple choice possible)	☐ 1<br>☐ 2<br>☐ 3<br>☐ 4<br>☐ 5<br>☐ 6<br>☐ 7<br>☐ 8<br>☐ 9<br>☐ 10<br>☐ 11<br>☐ 12<br>☐ 13<br>☐ other, namely:	yes
Welche beruflichen Qualifikationen haben Sie? (Mehrfachantwort möglich)<br>What professional qualifications do you have? (multiple answers possible)	☐ Geriatric nurse<br>☐ Paediatric nurse<br>☐ Nurse<br>☐ Bachelor Nurse<br>☐ Bachelor Nursing (dual) with professional license<br>☐ Bachelor Nursing without professional license<br>☐ Bachelor Public Health<br>☐ Bachelor Nursing Science<br>☐ Specialist training „school health professional“<br>☐ other, namely:	yes
The school(s) in which you work is/are located in a municipality/city with a population in the order of:<br>(multiple answers possible)	☐ more than 500.000<br>☐ 100.000 to 500.000<br>☐ 50.000 - less than 100.000<br>☐ 20.000 - less than 50.000<br>☐ 10.000 - less than 20.000<br>☐ 5.000 - less than 10.000<br>☐ less than 5.000	no
How many students are you responsible for as a school nurse? Please enter the number.	Number of students:	yes
What model of school nursing do you work to/how would you outline the purpose of your work as a school nurse? Please use the following field for your answer:	free text	no
Which areas of responsibility are part of your professional activity as a school nurse? (Multiple answers and free text possible)	☐ Collaboration with public health services	no
	☐ Networking with local stakeholders, namely	no
	☐ Screening (early detection)	no
	☐ Counselling	no
	☐ Pandemic support	no
	☐ Health promotion and situational and behavioural prevention in the school setting	no
	☐ Collaboration in strengthening health resources and competences (studentss, teachers, parents)	no
	☐ Acute health care	no
	☐ Nursing care/ assistance	no
	☐ Other tasks, namely:	no
As part of the study, we plan to conduct focus group discussions in addition to the survey and accompany school nurses in their daily work (observation). We also intend to test the developed training together with school nurses. If you are interested, we would be very pleased if you could provide us with your contact details (name and email address) for future contact. These details will be stored separately from your responses to the questions to ensure the survey remains anonymous.		no
We sincerely thank you for your time and for answering our questions! The questionnaire has now been saved. You can simply leave this page.		

Appendix 2. Interview guide focus groups*

Short introduction, and study information and Thank you. Information about the recording start.
Tasks what is known from the survey.
What are your activities, what is your range of tasks?
- How are your main tasks distributed?
- If you were to divide a month into a pie chart (cake), which task area would take up how much space? (If month is difficult, then school term).

What do you understand by health literacy/critical health literacy?
- What tasks do you carry out independently or in a supporting capacity in the area of increasing health competence?
- In which situations/on which occasions do you promote health literacy among students?
- with parents or teachers or colleagues?
- What tasks do you carry out independently or in a supporting capacity in the area of increasing critical health literacy?
- Are claims and misinformation an issue in your daily work with children, parents and teachers?
- As school nurse, how do you rate your own critical health literacy?
- What is your perception of how your offers are received? How do you determine this?

What are the processes like when you support lessons on a topic?
- Who has come up with the ideas/topics on which you support lessons?
- Who is asking whom?
- How confident do you feel with teaching?

Do you use existing teaching material / information material, for example from the BzgA?
- On which topics?
- What criteria do you use to select the materials?
- How do you like such materials?
- What would you wish for teaching materials?

What freedom do you have within the scope of your activities?
- spatial and temporal organisation?
- content?
- Are there any ideas that you have, which are unable to put into practice?
- If yes, what are they? Why do you think implementation is failing?

Which opportunity do you have to gain further qualifications according to your needs (financially and in terms of time)?
Which topics are of particular interest to you? Are they important to you in your work?
- What format should training programmes have so that they are compatible with your everyday working life? (Duration, e-learning, attendance, self-study?)
- Time frame, when should they take place (afternoons, weekends ...?)
- Are there any skills that you think you lack, but which would help you to perform your job even better? If yes, what are they?

Who do you interact with in the course of your work?
- What is the cooperation like with which professional groups?
- Who is authorised to issue instructions to you?
- Over whom do you exercise authority?

Regarding the range of tasks and the activities you carry out:
- What is facilitating?
- What inhibits you/are barriers?

Thank you. Do you have any questions or additional information, that we haven't discussed yet?
Thank you for your time and participation. I'll stop recording now.

Sociodemographic data focus group and observations*

How long have you been working as a school nurse?	☐ less than 1 year<br>☐ 1 - 5 years<br>☐ 6 - 10 years<br>☐ more than 10 years
Which age group do you categorise yourself in?	☐ younger than 25 years<br>☐ 25-35 years<br>☐ 36-45 years<br>☐ 46-55 years<br>☐ older than 55 years
Which gender do you identify with?	☐ female<br>☐ male<br>☐ inter*<br>☐ trans*, transman, transwoman<br>☐ An identity not mentioned here.<br>☐ I do not want to categorise myself by gender
In which federal state do you carry out your professional activity as a school nurse? (multiple answers possible)	☐ Baden-Wurttemberg<br>☐ Bavaria<br>☐ Berlin<br>☐ Brandenburg<br>☐ Bremen<br>☐ Hamburg<br>☐ Hesse<br>☐ Mecklenburg Western-Pomerania<br>☐ Lower Saxony<br>☐ Northrhine-Westphalia<br>☐ Rhineland-Palatinate<br>☐ Saarland<br>☐ Saxony<br>☐ Saxony-Anhalt<br>☐ Schleswig-Holstein<br>☐ Thuringia<br>☐ other, namely:
In how many schools do you work as a school nurse?	☐ 1<br>☐ 2<br>☐ more than 2, namely:<br>☐ no answer
The school(s) where you work is/are:<br>(multiple answers possible)	☐ School kindergarten/pre-school<br>☐ Primary school<br>☐ Gymnasium/grammar school<br>☐ Special school<br>☐ Integrated comprehensive school<br>☐ School with several educational programmes<br>☐ Secondary school<br>☐ International school<br>☐ other, namely:

Sociodemographic data focus group and observations* cont.

In which grades do you look after children?<br>(multiple answers possible)	☐ 1<br>☐ 2<br>☐ 3<br>☐ 4<br>☐ 5<br>☐ 6<br>☐ 7<br>☐ 8<br>☐ 9<br>☐ 10<br>☐ 11<br>☐ 12<br>☐ 13<br>☐ other, namely:
What professional qualifications do you have? (multiple answers possible)	☐ Geriatric nurse<br>☐ Paediatric nurse<br>☐ Nurse<br>☐ Bachelor Nursing (dual) with professional license<br>☐ Bachelor Nursing without professional license<br>☐ Bachelor Public Health<br>☐ Bachelor Nursing Science<br>☐ Specialist training „school health professional“<br>☐ other, namely:
The school(s) in which you work is/are located in a municipality/city with a population in the order of:<br>(multiple answers possible)	☐ more than 500.000<br>☐ 100.000 to 500.000<br>☐ 50.000 - less than 100.000<br>☐ 20.000 - less than 50.000<br>☐ 10.000 - less than 20.000<br>☐ 5.000 - less than 10.000<br>☐ less than5.000
How many students are you responsible for as a school nurse? Please enter the number	Number of students:

Appendix 3. Observation guide*

- What is the general daily routine like?
- What are your activities, what is your range of tasks?
- If possible, *observe one teaching unit* to support the teaching activities of the school.
- Interacting with the teaching staff and children present, focussing on the School Nurse, not students.
    - How is teaching/learning success monitored/measured?
    - What are the procedures when the School Nurse helps to organise lessons on a topic?
    - Who has come up with the ideas/topics on which the School Nurse helps to organise the lessons?
    - Who addresses whom?
    - How confident do school nurses feel when they (co-)organise lessons?
- Which *information materials* are used by the School Nurse?
    - o How do you select your materials?
    - o Do you have to comply with certain specifications? Who sets the specifications?
- What do you understand by health literacy/critical health literacy?
- What tasks do you carry out independently or supportively in the area of increasing health literacy?
- What do you notice where the students have the greatest deficits?
- What can the pupils perhaps already do quite well?
- What tasks do you carry out independently or with support in the area of increasing critical health literacy?
    - Can school nurses name tasks?
    - What do school nurses understand by critical health literacy?
    - How do school nurses assess their own critical health literacy?
- How does the school nurse *document* her work?
    - What does the school nurse document?
    - Is there a reporting obligation associated with this?
- - What freedom do school nurses have within the scope of their activities?
    - spatial and temporal organisation?
    - organisation of content?
- Are there ideas that school nurses have but cannot put into practice at this school?
    - If so, what is it?
    - Why does it fail?
- Do school nurses have the opportunity to gain further qualifications according to their needs (financially and in terms of time)?
- Are there any skills that you yourself lack that would help you to perform your professional activities even better?
    - If so, what is it?
    - What competences do you need?
- On the basis of which model is School Nursing carried out in the institution?
    - How can this be recognised in everyday life/work/collaboration?
- How can you recognise this in your everyday life/work/collaboration?
    - How are arrangements made?
    - What is the co-operation with which professional groups?
    - Who exercises authority over them?
    - Over whom do you exercise authority?
- Do you yourself see a discrepancy between the range of tasks described and the activities you carry out?
- What motivated you to become a school nurse?

Appendix 4. Interview guide expert interviews, example

**Aim of the project**	
So first would you explain your position and your work in the context of school nursing?	
- professional qualification,	
- professional activity,	- for how long<br>- what kind of schools<br>- age of the student at school
**Tasks**	
- What are school nurses tasks?	- prevention, health promotion
- What tasks do you carry out to increase health literacy?	- Is CHL a topic?
	- What is your understanding of HL?<br>- Critical Health Literacy
- In which situations do you address health literacy of student?	- Do you also address teachers or parents health literacy
**Teaching and lesson planning**	
- to what topics do you teach students?	- Alone or together with teacher?<br>- If not, is it no task for school nurses all over?
○ How do you prepare for this topics	○ What qualifications/special qualifications do you have?
○ If you give teaching lessons, how do you prepare them?	○ What resources do you use, how do you search?
**Collaboration and cooperation**	
- Please explain how you work together with community nurses.	- How do your tasks differ from each other?
	- Is there a cooperation for single cases, people, families?
	- How do you deal with questions of data protection?
- Who else do you interact with in the context of your work?	
- How are school nurses financed?	
Thank you. Do you have any questions or additional information, that we haven't discussed yet?	
Thank you for your time and participation. I'll stop recording now.	

Appendix 5. Final category sets

Table 1 Main categories focus groups, observations, open answers survey

**Category/**subcategory	**Category description/definition**	**Sample code**
**School nursing model**	The code is assigned for statements/open answers to the question: According to which model of school nursing do you work/how would you outline the objective of your work as a school nurse?	*"No direct model, but various models are incorporated into the work, such as salutogenesis, health promotion and prevention" (O2:37).*
**Taks**		
Health education	The code is assigned when it is stated that lessons are (co-)created. Includes subcodes for time, responsibility, subjects, ideas/initiation.	*„and then we have health education lessons in many different facets, which is also requested by the schools." (FG2: 10)*
Health promotion and prevention	The code is assigned when health promotion and prevention is mentioned/reported. This includes the subcategories health-promoting school and learning environment.	*"Working on the framework conditions of the school with regard to making the school more health-promoting" (FG1: 5)*
Pandemic support	The code is assigned for tasks related to the pandemic.	*Mask mandate, few wanted to disregard it, and those few kept them very busy. „A few can occupy you." (O5: 27)*
Strengthening health resources and competences	The code is assigned for tasks that relate to contribution in strengthening the health resources and competences/literacy of students, teachers and parents. The topics addressed are clustered as an extra category, as well as lesson design and project work.	*"Strengthening the health resources of the students, that's kind of a main focus, I would say that this is also somewhat the goal of our work." (FG1: 5)*
Screening	The code is assigned when school nurses report on the extent to which they carry out or support screening examinations.	*"And I do the screening with the KJGD in the third and sixth grades. It's a voluntary task, at least for us, in the sixth grade they don't even manage to screen the children from the KJGD when it gets to the seventh grade." (FG2: 14)*
Acute health care/First aid	The code is assigned on school nurses statements about the extent to which they carry out first aid measures.	*„We are also responsible for acute care, mean when children become acutely ill during the school day or have an accident" (FG2: 14)*
Nursing care/assistance	The code is assigned when school nurses perform medical/nursing tasks that go beyond first aid.	*"I don't know, one of you just said it too, yes, we have autism spectrum disorder, ADHD, which is constantly being adjusted or changed with medication. We also have to provide a lot of training in this area. Not just the parents, the teachers, so that they know about it or epilepsy. They are always very well adjusted, it happens very rarely, but when there is an epileptic seizure here at school, it's quite impressive for the teachers." (FG2: 37)*

Table 1 cont. Main categories focus groups, observations, open answers survey

Networking with local stakeholders	Actors with whom the school nurses are networked or are building a network are clustered into subcategories	*"I still miss our networking in the local neighbourhood a bit" (FG1: 28-29)*
Collaboration with public health services	The code is assigned for cooperation with the municipal public health services; other actors in the healthcare system are coded in the code 'Actors in the healthcare system'.	*"And yes, our cooperation with the public health service is different. Here in [place name], it works quite well. I work in the dental service, paediatric and youth health service together with the hygiene department.' (FG2: 14)*
Counselling	The code is assigned for statements on the extent to which they provide counselling, who they advise and how, subcategory counselling topics.	*"Trusted and contact person, so everything that is brought to me by teachers, participation assistants, counselling needs, or from parents, so right across the board" (FG2: 29)*
Others	The code is assigned for tasks that cannot be assigned to any other category.	*"it is also the administration" (FG1: 25)*
**Topics**	The code is assigned to the topics that school nurses address or offer to address as part of their (preventive) work and/or lesson organisation are coded under this code.	*"and apart from that, health education lessons are from A to Z. The things that are really requested. Like today, today it was the topic of grief." (FG2: 10)*
**School nurses' role and autonomy**	The code is assigned on how they understand their role and the associated challenges.	*"where we could then care for any injured children, so that's just not our area of responsibility" (FG1: 15)*
**Cooperation with other professions**	The code is assigned to statements about the professional groups with which the school nurses collaborate, as subcategories of how they estimate this collaboration and how agreements are made	*"I'm in contact with the social worker twice a week, oh nonsense, that's not true, I'm in contact with her at least once a day." (FG2: 149)*
**General conditions school nursing**	The code is awarded for the equipment of the school nurse, including the subcategories of financial resources, spatial resources, time resources.	*"I have 480 pupils at the school, it's not quite as big for me, I manage to go through the classes. That's why I do it." (Focus groups > FG2_Transcript: 47)*

Table 2 Main categories expert interviews with interestholder

Category	Category description/definition	Sample code
**Professional background**	The code is assigned for information on professional background, including professional qualifications and professional activity.	*"I'm a nurse. And I. I'm also have a profession as a school nurse." (E10: 6-8)*
**Relation to school nursing**	The code is assigned to statements about the interviewees' connection to school nursing.	*"At our school, the school nurse actually plays an important role from the very beginning, from the first grade onwards." (E7: 7)*
**Cooperation**	The code is assigned to statements on how cooperation with school nurses is organised. Subcategories students, parents, school staff, public health authority.	*"Maybe a social worker at a school or something. Yes, I can discuss one or two things with them. But I can't expect any medical or nursing feedback from him or her." (E1: 67)*
**Teaching**	The code is awarded for statements on lesson organisation.	*"I want to say that these possibilities of the school, of lesson organisation, are used in different ways, depending on how the schools structurally provide for this" (E1: 213)*
**Health literacy**	The code is awarded for statements on health competence/literacy.	*"And about health literacy, this is definitely a very important topic, in the field of activity of school health professionals, because we know about the connection between education and health. That both are interdependent, so to speak, and that higher educational status in many ways also leads to better health status. And that's why it's very important." (E3: 61-63)*
**General conditions school nursing**	The code is awarded for statements on the framework conditions of school nursing, subcategories include barriers, pilot projects, stabilisation, qualification, financing of school nurses.	*"It must also be said, we welcomed it and think it's the right thing to do. But these other structures you need your own office and you also need a waiting area, things like that, you have to have all that. (E1: 247)*
